# Low Intensity Pulsed Ultrasound Prevents Recurrent Ischemic Stroke in a Cerebral Ischemia/Reperfusion Injury Mouse Model via Brain-derived Neurotrophic Factor Induction

**DOI:** 10.3390/ijms20205169

**Published:** 2019-10-18

**Authors:** Cheng-Tien Wu, Ting-Hua Yang, Man-Chih Chen, Yao-Pang Chung, Siao-Syun Guan, Lin-Hwa Long, Shing-Hwa Liu, Chang-Mu Chen

**Affiliations:** 1 Department of Nutrition, China Medical University, Taichung 40402, Taiwan; ct-wu@mail.cmu.edu.tw; 2 Master Program of Food and Drug Safety, China Medical University, Taichung 40402, Taiwan; 3 Department of Otolaryngology, College of Medicine and Hospital, National Taiwan University, Taipei 10051, Taiwan; thyang37@ntu.edu.tw; 4 Institute of Toxicology, College of Medicine, National Taiwan University, Taipei 10051, Taiwan; r06447007@ntu.edu.tw (M.-C.C.); d04447003@ntu.edu.tw (Y.-P.C.); 5 Institute of Nuclear Energy Research, Atomic Energy Council, Taoyuan 32546, Taiwan; dangerous885036@gmail.com; 6 Division of Neurosurgery, Department of Surgery, College of Medicine and Hospital, National Taiwan University, Taipei 10051, Taiwan; linhua1976@yahoo.com.tw; 7 Department of Medical Research, China Medical University Hospital, China Medical University, Taichung 40402, Taiwan; 8 Department of Pediatrics, College of Medicine and Hospital, National Taiwan University, Taipei 10051, Taiwan

**Keywords:** apoptosis, brain-derived neurotrophic factor, ischemic stroke, low-intensity pulsed ultrasound, stroke recurrence

## Abstract

The incidence of stroke recurrence is still higher despite the advanced progression of therapeutic treatment and medical technology. Low intensity pulsed ultrasound (LIPUS) has been demonstrated to possess therapeutic effects on neuronal diseases and stroke via brain-derived neurotrophic factor (BDNF) induction. In this study, we hypothesized that LIPUS treatment possessed therapeutic benefits for the improvement of stroke recurrence. Adult male C57BL/6J mice were subjected to a middle cerebral artery occlusion (MCAO) surgery and then followed to secondary MCAO surgery as a stroke recurrence occurred after nine days from the first MCAO. LIPUS was administered continuously for nine days before secondary MCAO. LIPUS treatment not only decreased the mortality but also significantly moderated neuronal function injury including neurological score, motor activity, and brain pathological score in the recurrent stroke mice. Furthermore, the administration of LIPUS attenuated the apoptotic neuronal cells and increased Bax/Bcl-2 protein expression ratio and accelerated the expression of BDNF in the brain of the recurrent stroke mice. Taken together, these results demonstrate for the first time that LIPUS treatment arouses the expression of BDNF and possesses a therapeutic benefit for the improvement of stroke recurrence in a mouse model. The neuroprotective potential of LIPUS may provide a useful strategy for the prevention of a recurrent stroke.

## 1. Introduction

Stroke is the second leading cause of death and the third for disability. Globally, more than 30 million people had suffered the impacts of a stroke and associated disorders as reported in 2016 [1,2]. For three out of four patients a stroke is the first event, while one in four patients can experience a recurrent stroke [3]. Despite the decreased incident rate of ischemic stroke [4], the cumulative risk of stroke recurrence remains in need of a reduction. The recurrent events lead to a prolonged hospitalization, an elevated risk of morbidity and mortality, and the increased medical expenditure or burden [4]. Multi-factorial networks such as respiratory and cardiovascular disease [5,6], diabetes-associated disorders [7] as well as hyperlipidemia [8] potentially increase the risk of ischemic stroke and recurrence. Yamamoto and Bogousslavsky suggested that the coexistence of multiple etiologies plays a major role in stroke recurrence [9]. These complications of mechanisms appear to develop a potential therapeutic treatment or strategy for recurrent ischemic stroke.

Therapeutic ultrasound is commonly used in different medical or clinical areas including physical therapy [10], diagnosis and prevention of diseases, and the enhancement capacity of drug delivery via non-thermal and thermal effects [11,12]. Low-intensity pulsed ultrasound (LIPUS), a non-thermal type of therapeutically acoustic radiation, has been considered another choice for disease treatment such as in bone fracture healing [13]. The LIPUS procedure has been approved as a therapeutic model by the Food and Drug Administration [11]. Interestingly, LIPUS treatment has also been investigated and applied as a therapeutic treatment in the improvement of neuronal diseases or disorders in vitro and in vivo. Zhao et al. found that the protective effects of LIPUS treatment on the 1-Methyl-4-phenylpyridinium (MPP^+^)-induced neurotoxicity and mitochondrial dysfunction in PC12 cells [14]. Su et al. demonstrated that post-LIPUS treatment reduced brain edema and ameliorated the behavioral and pathological outcomes in a traumatic brain injury mice model [15]. Other investigators further demonstrated the importance of brain-derived neurotrophic factor (BDNF) induction after LIPUS treatment in neuronal injury models [16,17,18,19,20,21]. Liu et al. also found the positive effects of LIPUS treatment on ischemic stroke via BDNF induction in rat models [22]. These findings have indicated the potential therapeutic benefit of LIPUS treatment for BDNF induction on the protection of neuronal diseases, like stroke.

In this study, we hypothesized that consecutive LIPUS treatment possessed ameliorated effects on stroke recurrence via BDNF induction. A middle cerebral artery occlusion (MCAO)-induced recurrent stroke mouse model was performed to assess the effects of LIPUS on neurological function, pathological change, neuronal cell apoptosis, and BDNF induction in the brain.

## 2. Results

**Figure 1:**
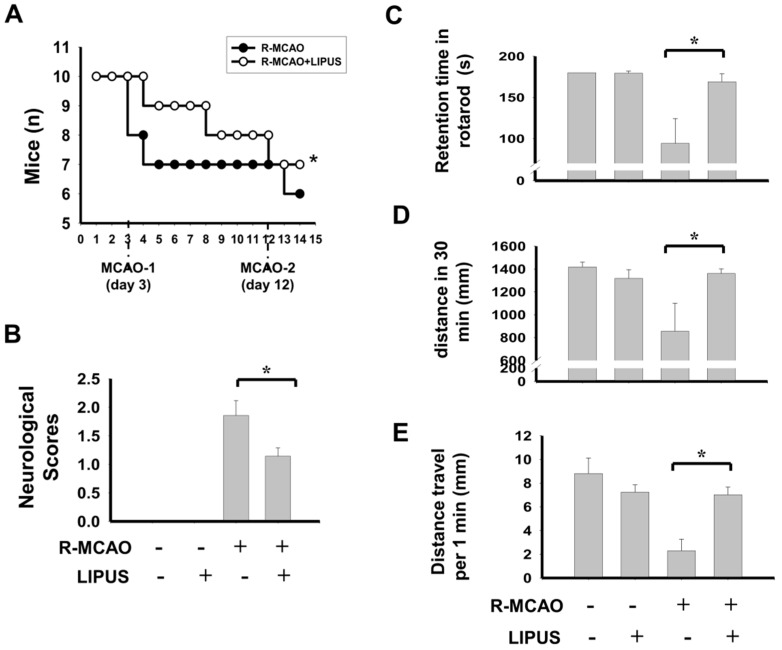
LIPUS treatment improved the neuronal functions in the recurrent stroke mice. Mice were treated with LIPUS 15 min daily for nine consecutive days before secondary MCAO procedure. (**A**) The survival mice were recorded from day 1 to day 14. A chi square Gehan–Breslow–Wilcoxon test was used for statistical analysis. * *p* = 0.017. (**B**) Neurological function scoring was evaluated at 24 h after a secondary MCAO procedure. Animal behavior tests were performed by a rotarod system (**C**) and a locomotor activity detection system including ambulation distance of locomotor activity (**D**) and average movement distance (**E**) after the next day of secondary MCAO procedure. Data are presented as mean ± SD (*n* = 10 per group). The ANOVA followed by the Bonferroni’s test was used for statistical analysis. * *p* < 0.05, versus R-MCAO group. L: LIPUS alone, R-MCAO: recurrent stroke model.

**Figure 2:**
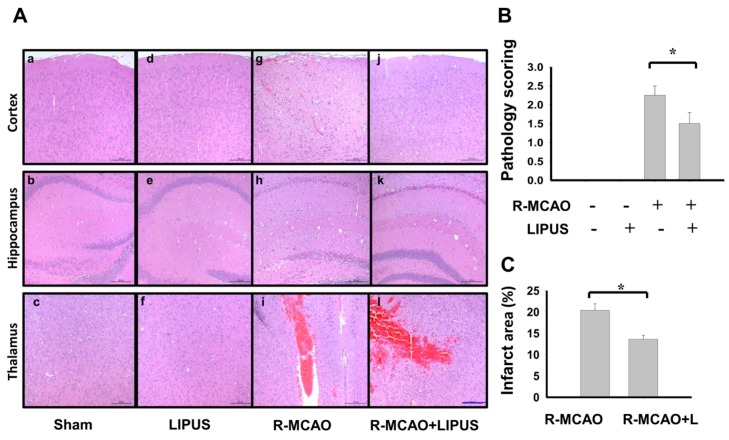
LIPUS treatment improved the pathological changes in the brains of the recurrent stroke mice. Mice were treated with LIPUS 15 min daily for nine consecutive days before a secondary MCAO procedure. The pathological changes in the left cerebral hemisphere tissues were detected by H&E staining after the secondary MCAO procedure. The cerebral cortex, hippocampus, and thalamus of each group were displayed (scale bar = 200 μm) (**A**). The pathological scoring is shown in (**B**). The infarct area is shown in (**C**). Data are presented as mean ± SD (*n* = 10 per group). The ANOVA followed by the Bonferroni’s test was used for statistical analysis. * *p* < 0.05, versus R-MCAO group. L: LIPUS alone, R-MCAO: recurrent stroke model.

In order to observe whether LIPUS treatment also ameliorated the neurological status of mice in a recurrent stroke condition, we first assessed the mortality and the changes in neuronal functions, which were performed by rotarod and locomotor activity tests. The parameters of LIPUS stimulation were performed as previously described [21]. As shown in Figure 1, LIPUS treatment displayed a lower mortality rate (Figure 1A; death/survival rate: day 4: MCAO, 8/10, MCAO+LIPUS, 10/10; day 8: MCAO, 7/10, MCAO+LIPUS, 9/10; day 14: Recurrent-MCAO, 6/10, Recurrent-MCAO+LIPUS, 7/10; *p* = 0.017), attenuated neurological deficit scores (Figure 1B; Recurrent-MCAO, 1.86 ± 0.26, Recurrent-MCAO+LIPUS, 1.14 ± 0.14, *n* = 10, *p* < 0.05), increased the retention time in a motor equilibrium performance on the rotarod (Figure 1C; Recurrent-MCAO, 94.29 ± 26.12 s, Recurrent-MCAO+LIPUS, 169.01 ± 9.76 s, *n* = 10, *p* < 0.05) and improved the loss of activity ability detecting by total distance (Figure 1D; Recurrent-MCAO, 855.55 ± 204.33 mm, Recurrent-MCAO+LIPUS, 1360.86 ± 40.45 mm, *n* = 10, *p* < 0.05) and movement rate (Figure 1E; Recurrent-MCAO, 2.30 ± 0.98 mm, Recurrent-MCAO+LIPUS, 7.02 ± 0.65 mm, *n* = 10, *p* < 0.05) in a recurrent stroke mouse model as compared to sham control mice. Moreover, the histopathological detection showed that moderate to severe locally extensive neuronal necrosis and neuronal cell loss with neuropil vacuolation, and hemorrhage were obviously observed in the hippocampus and cortex, and thalamus areas of recurrent stroke mice (Figure 2A). LIPUS administration conspicuously protected against these pathological changes (Figure 2A,B; pathological score: Recurrent-MCAO, 2.35 ± 0.25, Recurrent-MCAO+LIPUS, 1.50 ± 0.29, *n* = 10, *p* < 0.05). Similarly, LIPUS treatment significantly counteracted the increased infarction volume in recurrent stroke mice (Figure 2C; Recurrent-MCAO, 20.58 ± 1.56, Recurrent-MCAO+LIPUS, 13.29 ± 0.97, *n* = 10, *p* < 0.05). These results suggest that LIPUS treatment is capable of improving the pathological change and neuronal dysfunction of MCAO recurrent stroke mice.

**Figure 3:**
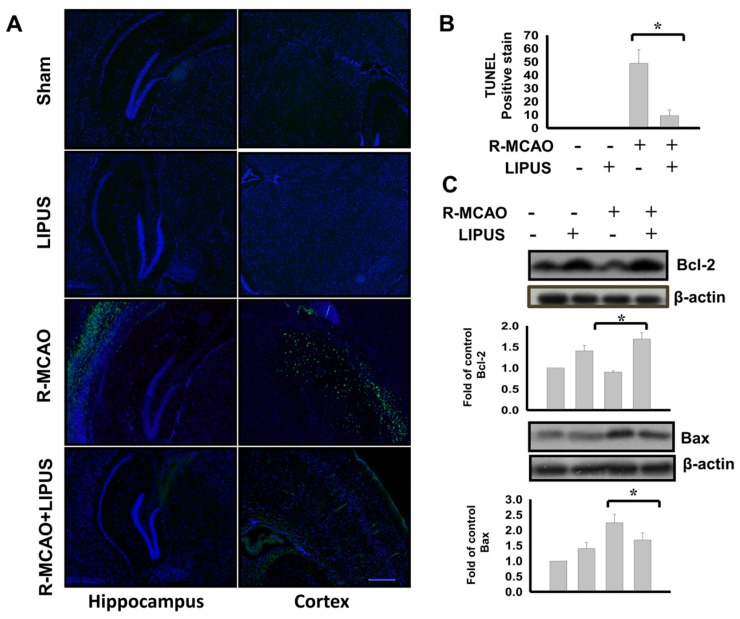
LIPUS treatment reduced the neuronal cell apoptosis in the left cerebral hemisphere tissues of the recurrent stroke mice. Mice were treated with LIPUS 15 min daily for nine consecutive days before the secondary MCAO procedure. The detection of neuronal apoptotic cells was performed by the TUNEL staining in the hippocampus and cortex. TUNEL-positive cells were presented as the fluorescent green color, while cell nuclei were displayed as the fluorescent blue color (scale bar = 200 μm) (**A**). The TUNEL-positive cells were counted and shown in (**B**). The protein expressions of Bax and Bcl-2 were determined by Western blotting in brain tissues (**C**). Data are presented as mean ± S.D. (*n* = 6 per group). The ANOVA followed by the Bonferroni’s test was used for statistical analysis. * *p* < 0.05, versus R-MCAO group. L: LIPUS alone, R-MCAO: recurrent stroke model.

We next tested the protective effect of LIPUS on neuronal cell apoptosis in recurrent stroke mice. As shown in Figure 3A,B, the TUNEL-positive cells for apoptotic neuron cells were not observed in the sham and LIPUS alone groups, however they were detected in the hippocampus and cortex of recurrent stroke mice. LIPUS treatment significantly decreased neuronal cell apoptosis in recurrent stroke mice (Figure 3A,B; apoptosis-positive staining: Recurrent-MCAO, 48.80 ± 10.21, Recurrent-MCAO+LIPUS, 9.40 ± 4.42, *n* = 6, *p* < 0.05). Moreover, the increased Bax protein expression and decreased Bcl-2 protein expression were observed in the left cerebral hemisphere tissues of recurrent stroke mice, which could be effectively reversed by LIPUS treatment (Figure 3C; Bcl-2: Recurrent-MCAO, 0.90 ± 0.03, Recurrent-MCAO+LIPUS, 1.69 ± 0.15 (fold of control), *n* = 6, *p* < 0.05; Bax: Recurrent-MCAO, 2.24 ± 0.18, Recurrent-MCAO+LIPUS, 1.68 ± 0.13 (fold of control), *n* = 6, *p* < 0.05). These results suggest that LIPUS treatment is capable of preventing neuronal apoptotic cell death in the brains of recurrent stroke mice.

**Figure 4:**
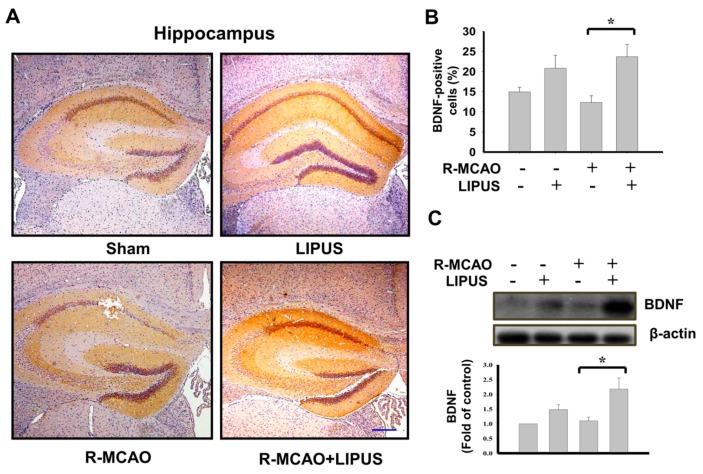
LIPUS treatment induced the protein expression of BDNF in the left cerebral hemisphere tissues of the recurrent stroke mice. Mice were treated with LIPUS 15 min daily for nine consecutive days before the secondary MCAO procedure. The protein expression of BDNF was determined by immunohistochemical staining (scale bar = 200 μm) (**A**,**B**) and Western blotting (**C**). The quantification for BDNF-positive cells (**B**) or BDNF protein expression (**C**) was shown. The BDNF-positive cells in the hippocampal CA1 regions were counted. Data are presented as mean ± SD (*n* = 6 per group). The ANOVA followed by the Bonferroni’s test was used for statistical analysis. * *p* < 0.05, versus R-MCAO group. L: LIPUS alone, R-MCAO: recurrent stroke model.

We next investigated whether LIPUS administration could improve the stroke recurrence through the induction of BDNF. As shown in Figure 4A, the representative images of BDNF immunohistochemical staining indicated that BDNF-positive cells broadly existed in the left cerebral hemispheres of sham mice, but sparsely scattered BDNF-positive cells were present in the cerebral cortex and hippocampus of the left cerebral hemisphere of recurrent MCAO mice. BDNF expression could be significantly and obviously induced in the hippocampus and cortex areas of the left cerebral hemisphere from recurrent stroke mice after LIPUS treatment. The quantification of BDNF-positive cells in hippocampus CA1 is shown in Figure 4B (Recurrent-MCAO, 12.31 ± 1.55, Recurrent-MCAO+LIPUS, 25.60 ± 1.94, *n* = 6, *p* < 0.05). Moreover, the protein expression of BDNF was also significantly elevated in the brain of recurrent stroke mice after LIPUS treatment (Figure 4B; BDNF: Recurrent-MCAO, 1.10 ± 0.12, Recurrent-MCAO+LIPUS, 2.18 ± 0.28 (fold of control), *n* = 6, *p* < 0.05). These results suggest that LIPUS treatment can moderate neuronal function loss and augment the expression of BDNF after recurrent stroke.

## 3. Discussion

Based on etiology, acute ischemic stroke was classified into five subgroups using the Trial of ORG 10,172 in the Acute Stroke Treatment Stroke (TOAST) classification, including large-artery atherosclerosis, small vessel occlusion, cardioembolism, stroke of other determined etiology, and stroke of undetermined etiology [23]. Chung et al. showed that large-artery atherosclerosis was the most common stroke subtype (37.3%) where the middle cerebral artery was the most frequently involved territory (49.6%), and suggested that the data acquisition of the vascular territory in a stroke lesion could help in timely survey and accurate diagnosis of stroke etiology [24]. Kocaman et al. found that 91 out of 500 ischemic stroke patients (18%) had recurrent ischemic stroke, and the risk factors of hypertension, diabetes, ischemic heart disease, hyperlipidemia, atrial fibrillation, and smoking which were found in 88%, 43%, 36%, 30%, 11%, and 14% of the patients, respectively. They also found that 38% of patients had more than two risk factors [25]. Recently, Yang et al. have reported that a pulmonary arteriovenous malformation is an overlooked cause in a patient with recurrent ischemic stroke, and suggested that a strategic protocol for searching the etiologies of cryptogenic stroke is imperative [26]. Recurrent stroke is a major risk factor for death and makes up approximately 15–25% of all the stroke patients that occur annually in the entire world [3,8]. Developing a new therapeutic window to improve the incidence rate of recurrent stroke is urgently needed. In the present study, the findings have demonstrated that LIPUS treatment arouses the expression of BDNF and possesses a therapeutic benefit for the alleviation of stroke recurrence in a mouse model.

The recurrent stroke animal model used in the current study was developed according to a previous study [27] with a slight modification. In our pilot test, we found that the lethal rate of mice was higher after MCAO treatment of more than 45 min and was harder to administer the subsequent LIPUS treatment and secondary MCAO (recurrence) surgical procedure. We, therefore, adjusted the cerebral artery occlusion duration for 30 min for two MCAO surgical procedures to avoid the heavy loading of mice. Moreover, the incidence rate of secondary stroke (recurrent stroke) has been reported to be approximately 10–20% within 90 days; some patients who have suffered an initial transient ischemic stroke may statistically display a higher risk of recurrence in the first two days after stroke [28,29,30]. Therefore, in the present study, the cerebral artery occlusion duration was 30 min for two MCAO procedures with a nine day interval between two MCAO procedures and the LIPUS treatment for nine days before the secondary MCAO procedure was considered to observe the best outcome in this recurrent stroke mouse model.

In the current study, we observed histopathological changes including locally extensive neuronal necrosis and neuronal cell loss with neuropil vacuolation and hemorrhage in the hippocampus, thalamus, and cortex areas of the recurrent stroke mice. As compared to our previous study [21] a hemorrhage was not observed in the hippocampus, thalamus, and cortex areas of the first ischemic stroke mouse model. Nevertheless, LIPUS effectively prevented these histopathological changes in the brains of first and recurrent stroke mice. Inflammation is known to play an important role in stroke events. A reduction in inflammation after stroke has been found to reduce brain ischemic injuries [31]. LIPUS treatment has also been shown to protect against ischemic injury in the brain via increased cerebral blood flow and decreased neutrophils [32]. Therefore, LIPUS may reduce the inflammatory response to alleviate brain ischemic injury in recurrent stroke mouse model.

We also observed increased TUNEL-positive cells in the hippocampus and cortex areas of recurrent stroke mice, indicating that apoptosis induction exists in neuronal cells. Cell apoptosis has been suggested to contribute to significant acute brain ischemia-induced neuron death, which apoptosis may initiate by some important molecular events in cells, including overproduction of free radicals, Ca^2+^ overload, and excitotoxicity [33]. Moreover, Korsmeyer et al. have shown that a pre-set ratio of Bcl-2/Bax determines the survival or death of cells exposed to the stimulation of apoptosis [34]. In the current study, we found that LIPUS stimulation significantly reversed the decreased Bcl-2 protein expression and increased Bax protein expression in the brain tissues of recurrent stroke mice, indicating that the increased Bcl-2/Bax ratio by LIPUS stimulation alleviated neuronal cell apoptosis.

BDNF is known to be a neurotrophic protective factor, involved in the regulation of several critical neuronal functions and neuroplasticity in patients who suffer nervous system diseases such as Parkinson’s disease, Alzheimer’s disease, and stroke [35,36,37]. Several empirical studies have indicated the benefits of the increased expression of BDNF or receptor in different stages of stroke such as acute ischemic stroke, the rehabilitation process, and recurrence [37,38,39,40]. The expression of BDNF has been demonstrated to have benefits on stroke recovery through several mechanisms and proposed actions including the increase in brain repair [7], the acceleration of neurogenesis [41], and the promotion of angiogenesis [42]. A previous study indicated that short-term memory impairment was closely associated with ischemia-induced apoptosis of hippocampal neuronal cells [43]. Liu et al. further indicated that neuronal cell proliferation and regeneration could be induced in the hippocampal dentate gyrus after transient global ischemia [44]. Neurogenesis has also been observed in the rat hippocampus after ischemic stroke [45]. Increased BDNF levels have been suggested to contribute to a compensatory response for neuronal regeneration in the rat brain after transient forebrain ischemia [46]. Previous in vivo studies have also indicated that LIPUS treatment can induce the expression of BDNF and alleviate Alzheimer’s disease [17] and neuronal degeneration of stroke-induced brain injury [21]. Similar to these previous studies [17,21], we also observed that BDNF was obviously induced in the hippocampus CA1 and dentate gyrus areas after LIPUS treatment in a recurrent ischemic stroke mouse mode.

Chen et al. have recently found that LIPUS treatment mitigates LPS-induced memory impairment, repression of inflammation as well as BDNF decline in an Alzheimer’s disease animal model via the modulation of TLR4/NF-κB signaling and CREB/BDNF expression [19]. LIPUS treatment has been shown to increase the expression of BDNF in astrocytes via the modulation of TrkB/PI3K/Akt and calcium/CaMK signaling pathways [18]. Our previous study also observed that LIPUS treatment effectively prevented the ischemia/reperfusion-induced cell apoptosis and injury in microglial cells via inducing BDNF protein expression [21]. However, the cellular sources of neuronal or non-neuronal cells (astrocyte and microglia) for BDNF observed changes in first and recurrent MCAO animal models with or without LIPUS stimulation requires further investigation in the future.

The efficacy of LIPUS on first ischemic stroke in a MCAO mouse model (one MCAO procedure) has been demonstrated in our previous study whereby LIPUS significantly ameliorates brain ischemic damage, displays a better neurological and behavior performance, decreases neuronal cell apoptosis, and upregulates brain BDNF expression [21]. Similarly, in the current study, we also found that LIPUS stimulation could effectively ameliorate neurological dysfunction and neuronal cell apoptosis through an upregulation of brain BDNF expression in a recurrent MCAO mouse model (two MCAO procedures). These findings suggest that LIPUS treatment not only ameliorates the neurological dysfunctions of a stroke (first event) but also alleviates stroke recurrence in a mouse model.

## 4. Materials and Methods

### 4.1. Animals and Middle Cerebral Artery Occlusion (MCAO) Recurrent Surgical Procedure

Six-week-old male C57 BL6J mice were obtained from the Animal Center, College of Medicine, National Taiwan University (Taipei, Taiwan). The Animal Research Committee of College of Medicine, National Taiwan University approved this animal study. The IACUC Approval Number is 20,180,385 (date of approval: 15th January 2019). During the experimental period, all experimental procedures and animal care were followed according to the guidelines of the Animal Research Committee of the College of Medicine, National Taiwan University and US-NIH for the welfare of the laboratory animals. Animals were humanely housed and fed in their cages at a constant temperature of 21 ± 2 °C and 12 h dark/light cycle. These animals were regarded for alleviation of suffering during surgery procedure. In this study, we completed a preliminary LIPUS parameters finding test (*n* = 3/group). Finally, considering 3Rs animal welfare, we used 10 male mice for each group and tested the therapeutic benefits of LIPUS in the formal experiments. Mice were randomly divided into four groups (*n* = 10), which were the Sham group, LIPUS treatment group, MCAO recurrence (R-MCAO) group, and MCAO recurrence combined LIPUS (R-MCAO+LIPUS) group. The MCAO procedure and recurrent operation were followed according to the previous studies by Hara et al. [47] and Qiao et al. [27], respectively, with slight modification. Briefly, for the MCAO operation, mice were anaesthetized using 2% isoflurane (Sigma-Aldrich, St. Louis, MO, USA) and maintained at a body temperature of 37 ± 0.5 °C with a hot plate. During neck dissection, the neck carotid bifurcation was detected to identify the left carotid artery as well as the external carotid artery. A thread with 6-0-nylon was used to insert the incision into the external carotid artery stump to the origin of the middle cerebral artery and the filament tip, which was coated with ethyl cyanoacrylate polymer, and occluded the middle cerebral artery for 30 min. Cerebral blood flow was monitored by a laser doppler detector (PeriFlux 4001, Perimed, Stockholm, Sweden). When the cerebral blood flow was reduced to less than 50% of the pre-ischemic condition, it was considered a standard procedure known as the Longa’s method [48]. For the recurrent stroke model, LIPUS treatment was applied for nine days after the first MCAO, and then a secondary MCAO surgical operation was carried out as the recurrent ischemic stroke procedure.

### 4.2. LIPUS Treatment

The setup of equipment and parameters for LIPUS were performed as previously described [21,49] with slight modification. Briefly, 1 MHz frequency of a single-focused transducer (A392S, Panametrics, Waltham, MA, USA) with a diameter of 38 mm and a radius of curvature of 63.5 mm was used for LIPUS treatment. 50% duty cycle and a repetition frequency of 1 Hz were applied. The transducer directly targeted the cortical of brain in MCAO treated mice by using a stereotaxic apparatus under an anesthesia condition. The acoustic power was performed with a 0.51 W (reflecting a spatial-peak temporal-average intensity of 528 mW/cm^2^) in the injured cortical areas 15 min daily for nine days before the secondary MCAO (recurrence) treatment. At the day of MCAO surgery and the next day, mice were resting and rehabilitated. All mice were assessed for their neurological functions, and then were necropsied for the detection of pathological changes on the final day.

### 4.3. Neurological Function Assessment

Neurological functions of MCAO recurrent stroke mice and control mice were estimated by the 4-point scale method, which was modified from Bederson’s neurological scoring [50], including 0: normal; 1, forelimb weakness and turn to the ipsilateral side when held by a tail; 2, circling to the contralateral side; 3, unable to bear weight on affected side; and 4, no spontaneous motor activity.

### 4.4. Locomotor Activity Evaluation

Locomotor activity was assessed in the specific-test boxes (9 × 20 × 11 cm) (Imetronic, Bordeaux, France) to evaluate the locomotor activity responses. Two lines of photocells in the boxes in which one was 2 cm above the floor to measure horizontal activity and the other was 6 cm above the floor to measure vertical activity (rears) with a low luminosity environment (5 lux). Mice were trained and stable in the testing box for 30 min before the formal experiment. The movement rate and total distance were recorded.

### 4.5. Assessment of Motor Equilibrium Performance on Rotarod

Motor equilibrium was assessed by a rotarod test as previously described [21]. Briefly, mice were trained for three days and tested through ten consecutive sections on the rotarod with a slow rotating speed (30 revolutions per min). When the cut off time point was reached (180 s), the retention time was recorded.

### 4.6. Infarct Volume Detection

Coronal slices of brain tissues were cut at 1–2 mm from the frontal tips. The sections were immersed in 2% 2,3,5-triphenyltetrazolium chloride at 37 °C for 20 min. The sections were then defined into areas, where areas that were not stained were considered as the infarction areas. The cerebral infarction areas were then calculated on the rostral and caudal surfaces of each section and the assessed infract volume was finally obtained and expressed as the percentage of the volume of the contralateral hemisphere. The measurement procedure was analyzed by the SigmaScan Pro 5.0 software (SPSS, Chicago, IL, USA).

### 4.7. Histopathological and Immunohistochemical Analysis

The brain tissues were isolated, fixed in the 10% formaldehyde buffer, and processed for the paraffin-embedded sections. Subsequently, 4–5 μm sections were stained by the hematoxylin and eosin (H&E) reagent. The degree of lesions were scored as previously described [51]. It was graded from one to five as follows: 1 = minimal (<1%); 2: slight (1–25%); 3 = moderate (26–50%); 4 = moderate/severe (51–75%); 5 = severe/high (76–100%). The pathological examination of brain tissues was performed by a veterinary pathologist with a blind test in the National Taiwan University College of Medicine Animal Center (Taipei, Taiwan). In addition, the immunohistochemical staining was performed as previously described [52]. Briefly, sections were deparaffinized and rehydrated by 95 to 50% ethanol and distilled water. The endogenous peroxidase activity was removed by the 3% hydrogen peroxide buffer and the non-specific antigen was blocked by the fetal bovine serum (FBS, 3%) after antigen retrieval. The primary antibody BDNF (Santa Cruz Biotechnology, Santa Cruz, CA, USA), Super Sensitive Polymer-HRP linker, 3,3′-diaminobenzidine tetrahydrochloride (DAB) detection system (BioGenex, CA, USA), and counter-stained agents (Sigma-Aldrich) were used.

### 4.8. Western Blotting

The brain tissues were dissected and homogenized in RIPA buffer (Sigma-Aldrich), pH 7.4. Equal amounts of total proteins were subjected to SDS-PAGE, transferred onto the PVDF membranes (Millipore Technology, Billerica, MA, USA), which were blocked with 5% bovine serum albumin (BSA; Sigma-Aldrich, USA), After blocking overnight, primary antibodies Bax, Bcl-2 (Cell Signal Technology, Danvers, MA, USA), BDNF, and β-actin (Santa Cruz Biotechnology) and the specificity secondary antibody were used. Finally, the membranes were detected by the chemiluminescence digital photo-image system (Thermo Fisher Scientific, Waltham, MA, USA) and digital quantification.

### 4.9. Fluorescent Terminal Deoxynucleotidyl Transferase (TdT) dUTP Nick End Labeling (TUNEL) Staining

Brain tissues were isolated and the paraffin-embedded sections were prepared. TUNEL staining was performed as previously described [53]. Briefly, 5 μm sections were heated for 30 min at 65 °C, the paraffin was removed in the xylene solution, and rehydrated in 90%, 75%, 50% ethanol for 5 min, respectively. After immersion in the distilled water and washing in the phosphate-buffer saline (PBS), the sections were stained by the Terminal deoxynucleotidyl transferase-mediated biotinylated UTP nick end labeling kit (TUNEL; Promega, Madison, WI, USA), and then the sections were stained by Hoechst 33,258 (1 mg/mL; Sigma-Aldrich) counter staining. The TUNEL-positive cells were directly counted in 10 randomly selected visual fields under 200× magnification.

### 4.10. Statistics

Data are presented as mean ± standard deviation (S.D.). The significant difference was analyzed by one-way analysis of variance (ANOVA) and followed by the Bonferroni’s test. For the statistical analysis of survival, the Chi square Gehan–Breslow–Wilcoxon Test was used. The *p* value < 0.05 was considered a statistically significant difference.

## 5. Conclusions

This study has demonstrated for the first time that consecutive administration of LIPUS displays a benefit for recurrent stroke via BDNF induction. The findings of our previous study [21] and the present study suggest that LIPUS treatment not only improves the neuronal dysfunctions of the stroke (first event) but also ameliorates stroke recurrence in a mouse model. The induction of BDNF may play an important role in the improvement of stroke and its recurrence after LIPUS treatment. LIPUS administration may serve as a potential therapeutic strategy for ischemic stroke and its recurrence.

## Abbreviations

LIPUS	Low intensity pulsed ultrasound
BDNF	Brain-derived neurotrophic factor
MCAO	Middle cerebral artery occlusion
TUNEL	Terminal deoxynucleotidyl transferase (TdT) dUTP nick end labeling

## References

[1] WHO The Top 10 Causes of Death. https://www.who.int/news-room/fact-sheets/detail/the-top-10-causes-of-death.

[2] Johnson W., Onuma O., Owolabi M., Sachdev S. (2016). Stroke: A global response is needed. Bull. World Health Organ..

[3] Bergstrom L., Irewall A.L., Soderstrom L., Ogren J., Laurell K., Mooe T. (2017). One-year incidence, time trends, and predictors of recurrent ischemic stroke in sweden From 1998 to 2010: An observational study. Stroke.

[4] Feigin V.L., Mensah G.A., Norrving B., Murray C.J., Roth G.A. (2015). Atlas of the global burden of stroke (1990–2013): The GBD 2013 Study. Neuroepidemiology.

[5] Siragusa S., Malato A., Saccullo G., Iorio A., Di Ianni M., Caracciolo C., Coco L.L., Raso S., Santoro M., Guarneri F.P. (2011). Residual vein thrombosis for assessing duration of anticoagulation after unprovoked deep vein thrombosis of the lower limbs: The extended DACUS study. Am. J. Hematol..

[6] Conte G., Rigon N., Perrone A., Lauro S. (1999). Acute cardiovascular diseases and respiratory sleep disorders. Minerva Cardioangiol..

[7] Tuttolomondo A., Maida C., Pinto A. (2015). Diabetic foot syndrome as a possible cardiovascular marker in diabetic patients. J. Diabetes Res..

[8] Oza R., Rundell K., Garcellano M. (2017). Recurrent ischemic stroke: Strategies for prevention. Am. Fam. Physician.

[9] Yamamoto H., Bogousslavsky J. (1998). Mechanisms of second and further strokes. J. Neurol. Neurosurg. Psychiatry.

[10] Ward R.S., Hayes-Lundy C., Reddy R., Brockway C., Mills P., Saffle J.R. (1994). Evaluation of topical therapeutic ultrasound to improve response to physical therapy and lessen scar contracture after burn injury. J. Burn Care Rehabil..

[11] Miller D.L., Smith N.B., Bailey M.R., Czarnota G.J., Hynynen K., Makin I.R. (2012). Overview of therapeutic ultrasound applications and safety considerations. J. Ultrasound Med..

[12] Piper R.J., Hughes M.A., Moran C.M., Kandasamy J. (2016). Focused ultrasound as a non-invasive intervention for neurological disease: A review. Br. J. Neurosurg..

[13] Lou S., Lv H., Li Z., Tang P., Wang Y. (2018). Effect of low-intensity pulsed ultrasound on distraction osteogenesis: A systematic review and meta-analysis of randomized controlled trials. J. Orthop. Surg. Res..

[14] Zhao L., Feng Y., Shi A., Zhang L., Guo S., Wan M. (2017). Neuroprotective effect of low-intensity pulsed ultrasound against MPP(+)-induced neurotoxicity in PC12 cells: Involvement of K2P channels and stretch-activated ion channels. Ultrasound Med. Biol..

[15] Su W.S., Wu C.H., Chen S.F., Yang F.Y. (2017). Low-intensity pulsed ultrasound improves behavioral and histological outcomes after experimental traumatic brain injury. Sci. Rep..

[16] Rodriguez-Frutos B., Otero-Ortega L., Ramos-Cejudo J., Martinez-Sanchez P., Barahona-Sanz I., Navarro-Hernanz T., Gomez-de Frutos Mdel C., Diez-Tejedor E., Gutierrez-Fernandez M. (2016). Enhanced brain-derived neurotrophic factor delivery by ultrasound and microbubbles promotes white matter repair after stroke. Biomaterials.

[17] Lin W.T., Chen R.C., Lu W.W., Liu S.H., Yang F.Y. (2015). Protective effects of low-intensity pulsed ultrasound on aluminum-induced cerebral damage in Alzheimer’s disease rat model. Sci. Rep..

[18] Liu S.H., Lai Y.L., Chen B.L., Yang F.Y. (2017). Ultrasound enhances the expression of brain-derived neurotrophic factor in astrocyte through activation of TrkB-Akt and calcium-CaMK signaling pathways. Cereb. Cortex.

[19] Chen T.T., Lan T.H., Yang F.Y. (2019). Low-intensity pulsed ultrasound attenuates LPS-induced neuroinflammation and memory impairment by modulation of TLR4/NF-κB signaling and CREB/BDNF expression. Cereb. Cortex.

[20] Li J., Zhang D.D., Wang C.Q., Shi M., Wang L.L. (2019). Protective effects of low-intensity pulsed ultrasound on aluminum overload-induced cerebral damage through epigenetic regulation of brain-derived neurotrophic factor expression. Biosci. Rep..

[21] Chen C.M., Wu C.T., Yang T.H., Liu S.H., Yang F.Y. (2018). Preventive effect of low intensity pulsed ultrasound against experimental cerebral ischemia/reperfusion injury via apoptosis reduction and brain-derived neurotrophic factor induction. Sci. Rep..

[22] Liu L., Du J., Zheng T., Hu S., Zhao M., Wang X., Wu S., Shi Q. (2019). Readout-segmented echo-planar diffusion-weighted MR at 3.0T for the evaluation the effect of low-intensity transcranial ultrasound on stroke in a rat model. Magn. Reson. Imaging.

[23] Adams H.P., Bendixen B.H., Kappelle L.J., Biller J., Love B.B., Gordon D.L., Marsh E.E. (1993). Classification of subtype of acute ischemic stroke. Definitions for use in a multicenter clinical trial. TOAST. Trial of ORG 10172 in Acute Stroke Treatment. Stroke.

[24] Chung J.W., Park S.H., Kim N., Kim W.J., Park J.H., Ko Y., Yang M.H., Jang M.S., Han M.K., Jung C. (2014). Trial of ORG 10172 in Acute Stroke Treatment (TOAST) classification and vascular territory of ischemic stroke lesions diagnosed by diffusion-weighted imaging. J. Am. Heart Assoc..

[25] Kocaman G., Dürüyen H., Koçer A., Asil T. (2015). Recurrent ischemic stroke characteristics and assessment of sufficiency of secondary stroke prevention. Noro Psikiyatr Ars.

[26] Yang X., Liu M., Zhu Y., Zhang X., Gao S., Ni J. (2018). An overlooked cause in a patient with recurrent ischemic stroke: A case report. Med. (Baltim.).

[27] Qiao M., Zhao Z., Barber P.A., Foniok T., Sun S., Tuor U.I. (2009). Development of a model of recurrent stroke consisting of a mild transient stroke followed by a second moderate stroke in rats. J. Neurosci. Methods.

[28] Hill M.D., Yiannakoulias N., Jeerakathil T., Tu J.V., Svenson L.W., Schopflocher D.P. (2004). The high risk of stroke immediately after transient ischemic attack: A population-based study. Neurology.

[29] Johnston D.C., Hill M.D. (2004). The patient with transient cerebral ischemia: A golden opportunity for stroke prevention. CMAJ.

[30] Nguyen-Huynh M.N., Johnston S.C. (2007). Evaluation and management of transient ischemic attack: An important component of stroke prevention. Nat. Clin. Pr. Cardiovasc Med..

[31] Chen J., Zhang X., Zhang C., Wang W., Chen R., Jiao H., Li L., Zhang L., Cui L. (2016). Anti-inflammation of natural components from medicinal plants at low concentrations in brain via inhibiting Neutrophil Infiltration after Stroke. Mediat. Inflamm..

[32] Guo T., Li H., Lv Y., Lu H., Niu J., Sun J., Yang G.Y., Ren C., Tong S. (2015). Pulsed transcranial ultrasound stimulation immediately after the ischemic brain injury is neuroprotective. IEEE Trans. Biomed. Eng..

[33] Radak D., Katsiki N., Resanovic I., Jovanovic A., Sudar-Milovanovic E., Zafirovic S., Mousad S.A., Isenovic E.R. (2017). Apoptosis and acute brain ischemia in ischemic stroke. Curr. Vasc. Pharm..

[34] Korsmeyer S.J., Shutter J.R., Veis D.J., Merry D.E., Oltvai Z.N. (1993). Bcl-2/Bax: A rheostat that regulates an anti-oxidant pathway and cell death. Semin. Cancer Biol..

[35] Li J., Zhang S., Li C., Li M., Ma L. (2018). Sitagliptin rescues memory deficits in Parkinsonian rats via upregulating BDNF to prevent neuron and dendritic spine loss. Neurol. Res..

[36] Bomba M., Granzotto A., Castelli V., Onofrj M., Lattanzio R., Cimini A., Sensi S.L. (2019). Exenatide reverts the high-fat-diet-induced impairment of BDNF signaling and inflammatory response in an animal model of Alzheimer’s disease. J. Alzheimers Dis..

[37] Luo L., Li C., Du X., Shi Q., Huang Q., Xu X., Wang Q. (2019). Effect of aerobic exercise on BDNF/proBDNF expression in the ischemic hippocampus and depression recovery of rats after stroke. Behav. Brain Res..

[38] Eleftheriou D., Ganesan V., Hong Y., Klein N.J., Brogan P.A. (2015). Endothelial repair in childhood arterial ischaemic stroke with cerebral arteriopathy. Cereb. Dis. Extra.

[39] Mang C.S., Campbell K.L., Ross C.J., Boyd L.A. (2013). Promoting neuroplasticity for motor rehabilitation after stroke: Considering the effects of aerobic exercise and genetic variation on brain-derived neurotrophic factor. Phys. Ther..

[40] Su Q., Cheng Y., Jin K., Cheng J., Lin Y., Lin Z., Wang L., Shao B. (2016). Estrogen therapy increases BDNF expression and improves post-stroke depression in ovariectomy-treated rats. Exp. Med..

[41] Schabitz W.R., Steigleder T., Cooper-Kuhn C.M., Schwab S., Sommer C., Schneider A., Kuhn H.G. (2007). Intravenous brain-derived neurotrophic factor enhances poststroke sensorimotor recovery and stimulates neurogenesis. Stroke.

[42] Kermani P., Hempstead B. (2007). Brain-derived neurotrophic factor: A newly described mediator of angiogenesis. Trends Cardiovasc Med..

[43] Ko I.G., Shin M.S., Kim B.K., Kim S.E., Sung Y.H., Kim T.S., Shin M.C., Cho H.J., Kim S.C., Kim S.H. (2009). Tadalafil improves short-term memory by suppressing ischemia-induced apoptosis of hippocampal neuronal cells in gerbils. Pharm. Biochem. Behav..

[44] Liu J., Solway K., Messing R.O., Sharp F.R. (1998). Increased neurogenesis in the dentate gyrus after transient global ischemia in gerbils. J. Neurosci..

[45] Yagita Y., Kitagawa K., Ohtsuki T., Takasawa K., Miyata T., Okano H., Hori M., Matsumoto M. (2001). Neurogenesis by progenitor cells in the ischemic adult rat hippocampus. Stroke.

[46] Tsukahara T., Iihara K., Hashimoto N., Nishijima T., Taniguchi T. (1998). Increases in levels of brain-derived neurotrophic factor mRNA and its promoters after transient forebrain ischemia in the rat brain. Neurochem. Int..

[47] Hara H., Huang P.L., Panahian N., Fishman M.C., Moskowitz M.A. (1996). Reduced brain edema and infarction volume in mice lacking the neuronal isoform of nitric oxide synthase after transient MCA occlusion. J. Cereb Blood Flow Metab..

[48] Ansari S., Azari H., McConnell D.J., Afzal A., Mocco J. (2011). Intraluminal middle cerebral artery occlusion (MCAO) model for ischemic stroke with laser doppler flowmetry guidance in mice. J. Vis. Exp..

[49] Yang F.Y., Lu W.W., Lin W.T., Chang C.W., Huang S.L. (2015). Enhancement of neurotrophic factors in astrocyte for neuroprotective effects in brain disorders using low-intensity pulsed ultrasound stimulation. Brain Stimul..

[50] Bederson J.B., Pitts L.H., Tsuji M., Nishimura M.C., Davis R.L., Bartkowski H. (1986). Rat middle cerebral artery occlusion: Evaluation of the model and development of a neurologic examination. Stroke.

[51] Shackelford C., Long G., Wolf J., Okerberg C., Herbert R. (2002). Qualitative and quantitative analysis of nonneoplastic lesions in toxicology studies. Toxicol. Pathol..

[52] Xiong J.Y., Li S.C., Sun Y.X., Zhang X.S., Dong Z.Z., Zhong P., Sun X.R. (2015). Long-term treadmill exercise improves spatial memory of male APPswe/PS1dE9 mice by regulation of BDNF expression and microglia activation. Biol. Sport.

[53] Chen C.M., Wu C.T., Chiang C.K., Liao B.W., Liu S.H. (2012). C/EBP homologous protein (CHOP) deficiency aggravates hippocampal cell apoptosis and impairs memory performance. Plos ONE.

